# Supplementary material for: Participation in Household Physical Activity Lowers Mortality Risk in Chinese Women and Men
Source: Int J Environ Res Public Health. 2023 Jan 5;20(2):987. doi: 10.3390/ijerph20020987 (PMC9859537; doi:10.3390/ijerph20020987)
Supplement: Supplementary file 1 [file ijerph-20-00987-s001.zip › ijerph-2112454-supplementary.pdf]

Supplementary Material

Participation in household physical activity lowers mortality risk in Chinese women and men

Lan Hu [1,2,†], Lu Wang [3,†], Yunquan Zhang [4,5,*], Ke Wang [1], Yaqi Wang [4], Huiyue Tan [4] and Yin Zhang [2,*]

1 Department of Nursing, Medical College, Wuhan University of Science and Technology, Wuhan 430065, China
2 Hubei Cancer Hospital, Tongji Medical College, Huazhong University of Science and Technology, Wuhan 430079, China
3 Department of Nursing, Wuhan 1st Hospital, Wuhan 430022, China
4 Department of Epidemiology and Biostatistics, School of Public Health, Wuhan University of Science and Technology, Wuhan 430065, China
5 Hubei Province Key Laboratory of Occupational Hazard Identification and Control, Wuhan University of Science and Technology, Wuhan 430065, China
* Correspondence: yunquanzhang@wust.edu.cn (Y.Z.); 13886180622@163.com (Y.Z.)
† These authors contributed equally to this work.

Contents

Table S1. Sensitive analysis of hazard ratios (95% CIs) for all-cause and cause-specific mortality, by excluding study participants with chronic diseases at baseline.

Table S2. Sensitive analysis of hazard ratios (95% CIs) for all-cause and cause-specific mortality, by excluding deaths in the first year of follow up.

Table S3. Sensitive analysis of hazard ratios (95% CIs) for all-cause and cause-specific mortality, by including the interviewing time.

Table S1. Sensitive analysis of hazard ratios (95% CIs) for all-cause and cause-specific mortality, by excluding study participants with chronic diseases at baseline.

Mortality outcome	HPA Per Day				SPE Per Day		
	0 hour	0—1 hour	1—3 hour	≥3 hour	0 min	1—60 min	≥60 min
**All-cause**							
Sex- and age- adjusted model	Ref.	0.55 (0.48—0.65)	0.52 (0.45—0.60)	0.41 (0.33—0.50)	Ref.	0.80 (0.69—0.91)	0.53 (0.38—0.74)
Multivariable-adjusted model [a]	Ref.	0.61 (0.52—0.72)	0.55 (0.47—0.65)	0.40 (0.32—0.51)	Ref.	0.95 (0.81—1.11)	0.56 (0.39—0.81)
**CVD**							
Sex- and age- adjusted model	Ref.	0.56 (0.36—0.89)	0.51 (0.33—0.79)	0.56 (0.32—0.98)	Ref.	0.96 (0.66—1.41)	0.64 (0.26—1.57)
Multivariable-adjusted model [a]	Ref.	0.68 (0.42—1.10)	0.58 (0.36—0.92)	0.51 (0.27—0.96)	Ref.	0.86 (0.55—1.34)	0.48 (0.19—1.23）
**RESP**							
Sex- and age- adjusted model	Ref.	0.44 (0.25—0.78)	0.33 (0.19—0.59)	0.17 (0.06—0.46)	Ref.	0.99 (0.60—1.63)	0.22 (0.03—1.59)
Multivariable-adjusted model [a]	Ref.	0.45 (0.23—0.85)	0.39 (0.20—0.73)	0.19 (0.06—0.56)	Ref.	1.17 (0.64—2.12)	0.26 (0.03,1.91)
**Cancer**							
Sex- and age-adjusted model	Ref.	0.65 (0.46—0.92)	0.77 (0.55—1.07)	0.53 (0.32—0.87)	Ref.	1.01 (0.76—1.35)	0.34 (0.13—0.93)
Multivariable-adjusted model [a]	Ref.	0.68 (0.47—0.97)	0.67 (0.47—0.95)	0.42 (0.24—0.72)	Ref.	1.11 (0.79—1.55)	0.41 (0.15—1.12)

[a]: We adjusted gender, age, BMI, ethnicity, marital status, residential region, geolocation, education attainment, employment status, household income, smoking status, alcohol consumption, sleep duration and chronic disease. Abbreviations: HPA, household physical activity; SPE, sport and physical exercise; CVD, cardiovascular disease; RESP, respiratory disease; BMI, body mass index; HR, hazard ratio; CI, confidence interval. Notes: we conducted sensitive analysis by excluding study participants with chronic diseases at baseline.

Table S2. Sensitive analysis of hazard ratios (95% CIs) for all-cause and cause-specific mortality, by excluding deaths in the first year of follow up.

Mortality outcome	HPA Per Day				SPE Per Day		
	0 hour	0—1 hour	1—3 hour	≥3 hour	0 min	1—60 min	≥60 min
**All-cause**							
Sex- and age- adjusted model	Ref.	0.52 (0.46—0.59)	0.47 (0.41—0.53)	0.39 (0.33—0.46)	Ref.	0.78 (0.69—0.88)	0.58 (0.44—0.76)
Multivariable-adjusted model [a]	Ref.	0.57 (0.50—0.66)	0.50 (0.44—0.58)	0.40 (0.33—0.48)	Ref.	0.91 (0.79—1.04)	0.57 (0.42—0.78)
**CVD**							
Sex- and age- adjusted model	Ref.	0.47 (0.33—0.69)	0.44 (0.31—0.64)	0.55 (0.36—0.86)	Ref.	0.85 (0.62—1.18)	0.64 (0.32—1.31)
Multivariable-adjusted model [a]	Ref.	0.58 (0.38—0.87)	0.50 (0.34—0.74)	0.51 (0.31—0.84)	Ref.	0.79 (0.54—1.15)	0.58 (0.27—1.22）
**RESP**							
Sex- and age- adjusted model	Ref.	0.58 (0.37—0.92)	0.39 (0.24—0.63)	0.32 (0.17—0.62)	Ref.	0.91 (0.61—1.37)	0.40 (0.13—1.25)
Multivariable-adjusted model [a]	Ref.	0.64 (0.39—1.07)	0.44 (0.26—0.74)	0.29 (0.14—0.63)	Ref.	1.16 (0.72—1.89)	0.54 (0.16—1.77)
**Cancer**							
Sex- and age-adjusted model	Ref.	0.61 (0.45—0.83)	0.69 (0.51—0.92)	0.60 (0.40—0.89)	Ref.	1.00 (0.77—1.28)	0.44 (0.21—0.94)
Multivariable-adjusted model [a]	Ref.	0.62 (0.45—0.85)	0.62 (0.45—0.84)	0.54 (0.35—0.83)	Ref.	1.08 (0.81—1.44)	0.51 (0.24—1.11)

[a]: We adjusted gender, age, BMI, ethnicity, marital status, residential region, geolocation, education attainment, employment status, household income, smoking status, alcohol consumption, sleep duration and chronic disease. Abbreviations: HPA, household physical activity; SPE, sport and physical exercise; CVD, cardiovascular disease; RESP, respiratory disease; BMI, body mass index; HR, hazard ratio; CI, confidence interval. Notes: we conducted sensitive analysis by excluding deaths in the first year of follow up.

Table S3. Sensitive analysis of hazard ratios (95% CIs) for all-cause and cause-specific mortality, by including the interviewing time.

Mortality outcome	HPA Per Day				SPE Per Day		
	0 hour	0—1 hour	1—3 hour	≥3 hour	0 min	1—60 min	≥60 min
**All-cause**							
Sex- and age- adjusted model	Ref.	0.52 (0.45—0.59)	0.46 (0.41—0.53)	0.39 (0.32—0.46)	Ref.	0.78 (0.69—0.88)	0.58 (0.44—0.76)
Multivariable-adjusted model [a]	Ref.	0.70 (0.61—0.81)	0.65 (0.56—0.74)	0.54 (0.45—0.66)	Ref.	0.81 (0.71—0.93)	0.56 (0.41—0.77)
**CVD**							
Sex- and age- adjusted model	Ref.	0.47 (0.33—0.69)	0.44 (0.31—0.64)	0.55 (0.36—0.86)	Ref.	0.85 (0.62—1.78)	0.64 (0.32—1.31)
Multivariable-adjusted model [a]	Ref.	0.65 (0.43—0.98)	0.58 (0.39—0.86)	0.64 (0.38—1.06)	Ref.	0.79 (0.54—1.15)	0.61 (0.29—1.29)
**RESP**							
Sex- and age- adjusted model	Ref.	0.58 (0.37—0.92)	0.39 (0.24—0.63)	0.32 (0.17—0.62)	Ref.	0.91 (0.61—1.37)	0.40 (0.13—1.25)
Multivariable-adjusted model [a]	Ref	0.82 (0.49—1.36)	0.60 (0.35—1.02)	0.41 (0.19—0.89)	Ref.	0.99 (0.61—1.59.)	0.54 (0.16—1.79)
**Cancer**							
Sex- and age-adjusted model	Ref.	0.61 (0.33—0.69)	0.69 (0.31—0.64)	0.60 (0.36—0.86)	Ref.	1.00 (0.77—1.28)	0.44 (0.21—0.94)
Multivariable-adjusted model [a]	Ref.	0.74 (0.54—1.02)	0.79 (0.58—1.08)	0.73 (0.48—1.12)	Ref.	0.95 (0.71—1.26)	0.51 (0.23—1.10)

[a]: We adjusted gender, age, BMI, ethnicity, marital status, residential region, geolocation, education attainment, employment status, household income, smoking status, alcohol consumption, sleep duration, chronic disease and interviewing time. Abbreviations: HPA, household physical activity; SPE, sport and physical exercise; CVD, cardiovascular disease; RESP, respiratory disease; BMI, body mass index; HR, hazard ratio; CI, confidence interval. Notes: we conducted sensitive analysis by including the interviewing time.
